# Aggregation and Cellular Toxicity of Pathogenic or Non-pathogenic Proteins

**DOI:** 10.1038/s41598-020-62062-3

**Published:** 2020-03-20

**Authors:** Sungmun Lee, Myung Chul Choi, Kenana Al Adem, Suryani Lukman, Tae-Yeon Kim

**Affiliations:** 1 Department of Biomedical Engineering and Healthcare Engineering Innovation Center, Khalifa University of Science and Technology, PO Box 127788, Abu Dhabi, United Arab Emirates; 2 Department of Bio and Brain Engineering, Korea Advanced Institute of Science and Technology (KAIST), Daejeon, 34141 South Korea; 3 Department of Chemistry, College of Arts and Science, Khalifa University of Science and Technology, P.O. Box 127788, Abu Dhabi, United Arab Emirates; 4 Department of Civil Infrastructure and Environmental Engineering, Khalifa University of Science and Technology, P.O. Box 127788, Abu Dhabi, United Arab Emirates

**Keywords:** Cellular neuroscience, Neurodegeneration

## Abstract

More than 20 unique diseases such as diabetes, Alzheimer’s disease, Parkinson’s disease are caused by the abnormal aggregations of pathogenic proteins such as amylin, β-amyloid (Aβ), and α-synuclein. All pathogenic proteins differ from each other in biological function, primary sequences, and morphologies; however, the proteins are toxic when aggregated. Here, we investigated the cellular toxicity of pathogenic or non-pathogenic protein aggregates. In this study, six proteins were selected and they were incubated at acid pH and high temperature. The aggregation kinetic and cellular toxicity of protein species with time were characterized. Three non-pathogenic proteins, bovine serum albumin (BSA), catalase, and pepsin at pH 2 and 65 °C were stable in protein structure and non-toxic at a lower concentration of 1 mg/mL. They formed aggregates at a higher concentration of 20 mg/mL with time and they induced the toxicity in short incubation time points, 10 min and 20 min only and they became non-toxic after 30 min. Other three pathogenic proteins, lysozyme, superoxide dismutase (SOD), and insulin, also produced the aggregates with time and they caused cytotoxicity at both 1 mg/mL and 20 mg/mL after 10 min. TEM images and DSC analysis demonstrated that fibrils or aggregates at 1 mg/mL induced cellular toxicity due to low thermal stability. In DSC data, fibrils or aggregates of pathogenic proteins had low thermal transition compared to fresh samples. The results provide useful information to understand the aggregation and cellular toxicity of pathogenic and non-pathogenic proteins.

## Introduction

More than 20 different human diseases such as Alzheimer’s disease, Parkinson’s disease, and type II diabetes are associated with protein misfolding and aggregation^1,2^. Misfolded or unfolded proteins under unfavorable environmental conditions are prone to form aggregates via hydrophobic interactions of proteins^1,3,4^. Soluble proteins are converted into insoluble aggregates that cause cellular toxicity^5^. In amyloidosis, insoluble protein aggregates are deposited extracellularly and they leads to tissue damage and diseases^6^. Pathogenic proteins in amyloidosis include lysozyme, Aβ, insulin, superoxide dismutase (SOD), α-synuclein, amylin, and β2-macroglobulin^7–9^. Although all proteins differ from each other in biological function, primary sequences, and morphologies, pathogenic proteins aggregates share many common features such as insolubility, protease resistance, and optical behavior with certain dyes such as thioflavin T (ThT)^10,11^.

Human body generates many different kinds of proteins. According to the Human Proteome Project (HPP), the database has 19,467 predicted protein-coding genes^12^. Other databases such as the Human Protein Atlas have antibody findings for more than 12,000 protein-coding genes^13^. Although there are lots of proteins in the human body, not all of human proteins are amyloidogenic or pathogenic. Protein folding and aggregation are affected by various factors such as isoelectric point (pI), pH, ionic strength, temperature, protein concentration and the secondary structure of proteins^14^. The isoelectric point (pI) is the pH of a solution at which the net charge of a protein becomes zero. Protein solubility is minimal at its pI due to minimal protein charge–charge repulsions. Amyloid-like fibril formation is often seen at pH values away from PI^15^. Ionic strength and pH are other key solution conditions, which alter charge–charge interactions. High temperatures disrupt hydrogen bonding and hydrophobic interactions of proteins, which leads to protein denaturation and aggregation. The effect of protein concentration on protein aggregation has been investigated extensively^14^. The increase in protein concentration enhances the formation of protein aggregates due to increased chance of collision^16^. The secondary structure transition from α-helices to β-sheets also precedes aggregation of proteins. In this research, we selected six proteins, bovine serum albumin (BSA), catalase, pepsin, insulin, SOD, and lysozyme, and we investigated similarities and differences in protein aggregation kinetics, cellular toxicity, and morphological structures of six proteins.

Here is the basic information of six proteins that are selected for this research. This is first step to understand the protein aggregation of proteins.

Albumin is the most abundant protein in plasma. Bovine serum albumin (BSA) is a globular protein and biological function of BSA includes the delivery of nutrients to cells, balancing plasma pH, and solubilization of fatty acids. Due to its stability, BSA is used for numerous biochemical applications such as drug delivery systems^17^. BSA molecule consists of 583 amino acids and the molecular weight of BSA is 66.4 kDa^18^. The amino acid chain is made up of three homologous domains (I, II and III) and each domain consists of two sub-domains, A and B^19,20^. Secondary structure is important for enzymatic function and protein aggregation. The secondary structure of BSA is mainly α-helical (74%) and the remaining polypeptide chain is in turns^19,21^. BSA has abundant ionic amino acids such as glutamic acid and lysine, while it has low tryptophan, methionine, glycine and isoleucine content. The isoelectric point of bovine serum albumin is 4.8^21^.

Catalase (EC 1.11.1.6) is an antioxidant enzyme that decomposes hydrogen peroxide to water and oxygen^22–24^. The beef liver catalase monomer consists of 506 amino acids with one heme group and one NADH molecule. Catalase is a tetramer of four identical holo monomers^25^. Each monomer has 26.4% α-helices and 21.7% β-sheet in secondary structure^26^. Irregular structure including single stands and loops plays a major role in the assembly of the tetramer. Isoelectric point of the protein is 6.7^27^.

Pepsin (EC 3.4.23.1) is an enzyme that breaks down proteins into smaller peptides. It is one of the main digestive enzymes produced in the stomach. The maximal optimal pH for the pepsin is 1.5–2.5^28^. Porcine pepsin consists of 327 amino acids, which has a molecular weight of 34,620 g/mol^29^. Isoelectric point of the porcine pepsin is between 2.76 and 2.90^30^. The secondary structure of pepsin is 14% helices and 44% β-sheet. Ten α-helices encompasses 46 residues and 32 β-strands encompasses 144 residues^31^. There are random coils as well.

Superoxide dismutase (SOD, EC 1.15.1.1) is an enzyme that converts superoxide (O_2_·^−^) radical into oxygen (O_2_) and hydrogen peroxide (H_2_O_2_)^32^. There are three isoforms, known as SOD1, SOD2, and SOD3^33^. SOD1 is the cytosolic copper (Cu)-zinc (Zn) dimeric form, SOD2 is the mitochondrial manganese SOD tetrameric form, and SOD3 is the extracellular Cu, Zn tetrameric form. Mutations of SOD1 gene have been associated with amyotrophic lateral sclerosis (ALS), also known as Lou Gehrig’s disease^34^. SOD1 has a molecular weight of 32 kDa with one copper- and one zinc-binding site per 153-amino acid subunit. SOD2 and SDP3 have molecular weights of 92 kDa and 135 kDa respectively^33^. The secondary structure of SOD1 is 4.4% helices and 29.9% β sheets^35^. The isoelectric point of human SOD1 is 6.3, 6.0, 5.7, and 5.0 for four isoforms^36^.

Insulin is a hormone that allows the blood glucose to enter cells and a deficiency of the insulin or inefficient use of the insulin causes diabetes^37^. Insulin is a globular protein containing two chains, A (21 residues) and B (30 residues)^38,39^. The A chain contains an N-terminal α-helix (residues A1-A8), turn in the middle, and C-terminal helix (A12-A19). The B chain contains an N-terminal strand (B1-B5), β-turn (B6-B9), central α − helix (B9-B19), second β-turn (B20-B23), and C-terminal β-strand (B24-B28)^40^.

Lysozymes (EC 3.2.1.17) are a family of enzymes with antimicrobial activity by breaking down bacterial cell walls^41^. Mutations in human lysozyme can cause protein misfolding and large quantities of lysozyme aggregates can be accumulated in liver, kidney, and other regions of gastrointestinal tract^42,43^. Due to lots of information available on lysozyme aggregation coupled with the multiple conditions, lysozyme has been used as a model protein for amyloid fibril formation^9^. Hen egg white lysozyme (HEWL) is homologous to human lysozyme that is responsible for non-neuropathic systemic amyloidosis and the aggregation of HEWL stimulates cellular toxicity. HEWL is a single polypeptide chain and the molecular weight of HEWL with 129 amino acids is 14.3 kDa^44^. HEWL has four intramolecular disulfide bridges and an isoelectric point near 11.35. HEWL has four α-helices, a 3_10_-helix, and a triple-stranded anti-parallel β-sheet with four disulphide bonds^45^. The lysozyme aggregation has been studied extensively *in vitro*. Since there is no net charge of lysozyme near isoelectric point, pH 11.35, lysozyme precipitation was observed over pH 11. For example, HEWL (4–200 μM) at pH 12.2 aggregates without a lag phase at room temperature (25 °C)^46^. HEWL also forms fibrils in acidic pHs. HEWL was incubated at either 37 °C or 65 °C with either pH 2.0 or 4.0^42^. Fibril formation was observed in all conditions; however, the fastest rate of fibril formation was at 65 °C with pH 2. HEWL above the critical concentration, 50 μM, also aggregates at pH 1.6 and 65 °C. Protein denaturants such as ethanol and guanidine hydrochloride were also used for lysozyme aggregation study. HEWL can serve as a model protein to investigate lysozyme amyloidosis.

Proteins are susceptible to environmental changes such as temperature, pH, and denaturants^47^. Unfolded proteins induced by unfavorable environments are prone to form aggregates via hydrophobic interaction between proteins. At pH 2, hen egg white lysozyme (HEWL) aggregates faster than other acidic pHs such as pH 3 and pH 4^42^. The aggregation process of HEWL can be accelerated when the protein is at higher temperatures (e.g. 60 °C, 65 °C, and 80 °C)^42^. The aggregation kinetics also depends on the protein concentration. Higher concentration (3.0 wt %) of HEWL reduces the lag time in protein aggregation than lower concentrations (1.0 wt % or 2.0 wt %), and higher concentration also enhances the light scattering intensity^42^. In this research, we dissolve six proteins, BSA, catalase, pepsin, insulin, SOD, and lysozyme in pH 2 solution with 150 mM NaCl and we incubate them at 65 °C. In order to understand the effect of protein concentration on the kinetics of protein aggregation, two concentrations (1 mg/mL and 20 mg/mL) of proteins are selected. We will investigate protein aggregation kinetics, cellular toxicity, and morphological structure of six proteins in this condition.

## Materials and Methods

### Materials

All six proteins, bovine serum albumin (BSA), catalase, pepsin, lysozyme, insulin, and superoxide dismutase (SOD), were purchased from Sigma-Aldrich (Saint Louis, MO, USA). All other chemicals, unless otherwise specified, were obtained from Sigma-Aldrich.

### Protein sample preparation

Lyophilized proteins (1.0 mg or 20.0 mg) were freshly dissolved in 1.0 mL of pH 2 solution with 150 mM NaCl. The solution in 1.5 mL eppendorf tubes was incubated at 65 °C under a quiescent condition and samples were taken out at designated time points. For cell viability assays, RPMI 1640 medium adjusted to pH 2 was used instead of pH 2 solution.

### Thioflavin-T (ThT) fluorescence assay

Thioflavin T, a benzothiazole salt, is widely used to quantify the protein aggregates. Protein samples (80 μL) was mixed with 80 µL of ThT solution (30 µM) in a 96-well plate. After 20 minute incubation, the ThT fluorescence intensity of each sample was measured by excitation at 440 nm and emission at 490 nm, using an Infinite® 200 Pro microplate reader (Tecan Trading AG, Switzerland).

### 8-Anilinonaphthalene-1-sulfonic acid (ANS) fluorescence assay

ANS (Sigma-Aldrich, Saint Louis, MO, USA) was dissolved at 0.1% w/v in Na-K phosphate buffer (8 mM Na2HPO4, 2 mM KH2PO4, 140 mM NaCl, pH 7.2). Protein samples (140 µL) were incubated with 15 µL of ANS stock solution and the fluorescence of ANS was excited at 385 nm and the emission intensities were observed at 500 nm. A 20 μL sample was also withdrawn for protein concentration determination using the Bradford protein assay.

### Cell culture

PC-12 (ATCC^®^ CRL-1721^™^) cell lines from the American Type Culture Collection (ATCC) (Manassas, VA) were maintained at 37 °C under a humidified atmosphere of 5% CO_2._ PC-12 cells were maintained in RPMI-1640 medium containing 5% (v/v) fetal bovine serum and 10% (v/v) heat-inactivated horse serum, supplemented with 1% penicillin (100 U/mL) and streptomycin (100 µg/mL).

### Cytotoxicity of protein aggregates

An MTT (3-(4,5-Dimethylthiazol-2-yl)-2,5-diphenyltetrazolium bromide) reduction assay was performed to measure the toxicity of protein aggregates. Protein samples were prepared as described in the protein sample aggregation section. At designated time points (10 min, 20 min, 30 min, and 60 min), protein samples were neutralized to pH 7.4 and they were added to PC-12 cells (2 × 10^4^ cells/well in 96 well plates) in RPMI 1640. The cells were treated with protein samples for 24 hours followed by the MTT assay. Next, 10 μL of MTT solution (5 mg/mL in PBS) was added to each well, and the cells were incubated for 4 hours. Yellow MTT solution is reduced to purple formazan in living cells and the absorbance of formazan crystals was measured at 570 nm (λ_abs_ = 570 nm) using an Infinite® 200 Pro microplate reader. Percentage cell viability was calculated by comparing the absorbance of the control cells (cells with medium only without any protein aggregates) to that of protein aggregates-treated cells (sample). Percentage cell viability = (sample with MTT – sample)/(control with MTT – control) × 100.

### Transmission electron micrograph (TEM)

Six proteins, BSA, catalase, pepsin, lysozyme, insulin, and SOD, in pH 2 solution (1.0 mg/mL) were incubated at 65 °C for 2 hours. Then, 20 μL of protein solution were placed on carbon-coated copper grids (Ted-Pella Inc, Redding, CA, USA), and the protein samples on the grid were negatively stained with 2% aqueous ammonium molybdate. After sufficient air-dry, grids were examined in a FEI Tecnai transmission electron microscope (TEM) at an accelerating voltage of 200 kV.

### Circular dichroism (CD)

Six proteins, BSA, catalase, pepsin, lysozyme, insulin, and SOD, in pH 2 solution (1.0 mg/mL) were incubated at 65 °C for 2 hours. Secondary structure of fresh proteins at pH 7.0 or proteins at pH 2.0 for 2 hours was measured using a Jasco model J-815 circular dichroism (CD) spectropolarimeter (Jasco, Tokyo, Japan). CD spectra in the far UV range (190–260 nm) were obtained using a 2 mm quartz cell and a Xe lamp as a light source. A deconvolution method (BeStSel) of CD spectrum was used to estimate the secondary structures of fresh proteins and protein aggregates at pH 2.0 for 2 hours^48^.

### Statistics

Each bar graph represents a mean ± standard deviation of at least three independent experiments. Statistical analysis was performed using Student’s t-test, comparing each treatment to cells only unless otherwise mentioned. In all statistical analysis p < 0.05 (*) was considered significant.

## Results and Discussion

Protein may cause cellular toxicity when aggregated. Fibrillar aggregates of proteins are associated with more than 20 degenerative diseases such as Alzheimer’s disease, Parkinson’s disease, and type II diabetes respectively^1,2^. Protein aggregation is influenced by physicochemical and biological factors such as temperature, pH, and salt concentration^49^. However, what leads to protein toxicity has not been clearly elucidated.

Six proteins, lysozyme, insulin, superoxide dismutase (SOD), bovine serum albumin (BSA), catalase, and pepsin, were selected and similarities and differences in aggregation mechanisms were investigated in this research. Three proteins, lysozyme, insulin, superoxide dismutase (SOD), were in the list of pathogenic proteins. Catalase, an antioxidant enzyme, was selected as a counterpart of SOD, another antioxidant enzyme. Pepsin, a digestive enzyme, was chosen as a counterpart of lysozyme, an antimicrobial enzyme. BSA, the most abundant protein in plasma, was used for a counterpart of insulin, a hormone that regulates the blood glucose level.

**Figure 1:**
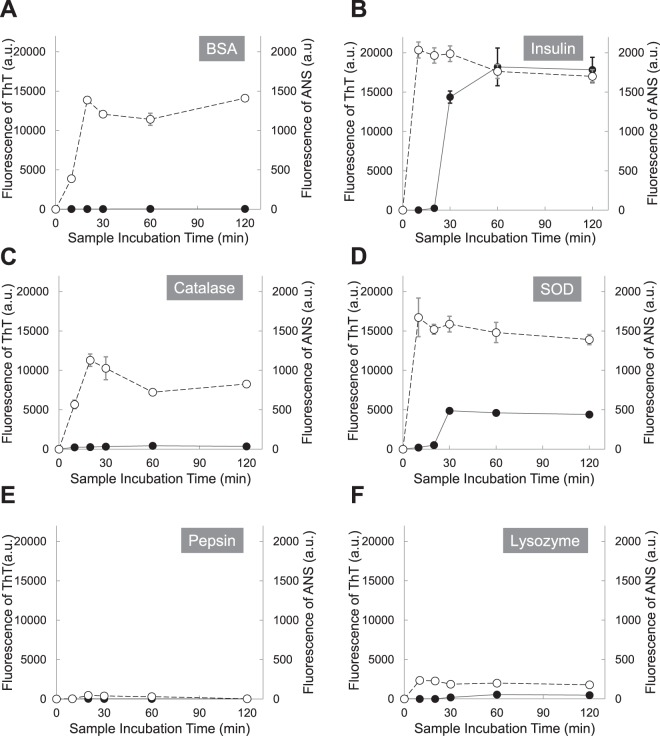
Protein (1 mg/mL) aggregation kinetics at pH 2 (150 mM NaCl) and 65 °C was monitored by fluorescence of Thioflavin T (ThT) and 8-Anilinonaphthalene-1-sulfonic acid (ANS). ThT fluorescence (black circles) and ANS fluorescence (white circles); (**A**) BSA, (**B**) Insulin, (**C**) Catalase, (**D**) Superoxide dismutase (SOD), (**E**) Pepsin, and (**F**) Lysozyme.

In Fig. 1, six proteins (1 mg/mL) were incubated at pH 2 with 150 mM NaCl and 65 °C, and the aliquots were withdrawn at the following time points (0, 10, 20, 30, 60 and 120 minutes). Aggregation patterns were monitored by fluorescence of Thioflavin T (ThT) and 8-Anilinonaphthalene-1-sulfonic acid (ANS). Apparently all proteins seem to follow a nucleation-dependent aggregation model. However, three proteins, BSA, catalase, and pepsin, increased ThT fluorescence emission without a lag time and they reached the saturation level in 30 minutes. The ThT fluorescence differences between fresh and aged samples of BSA, catalase, and pepsin, were small compared to other three proteins. The intensity differences of ThT fluorescence between 0 minute sample and 30 minute sample were 28.7 ± 3.1 a.u (mean ± standard deviation) for BSA, 319.0 ± 41.4 a.u for catalase, and 12.7 ± 9.1 a.u for pepsin. Other three proteins, lysozyme, insulin, and SOD, increased ThT fluorescence emission with 10 minute lag time and they reached the saturation level in 30–60 minutes. The intensity differences of ThT fluorescence between 0 minute sample and 60 minute sample were 14,360.0 ± 778.8 a.u for insulin, 4,869.3 ± 166.3 a.u for SOD, and 546.7 ± 60.3 a.u for pepsin. The ANS fluorescence of all proteins was increased in 10 minutes and a plateau was reached after 10 minutes. Lysozyme, insulin, and SOD, reached a plateau slightly faster than BSA, catalase, and pepsin. Unlike other proteins, pepsin had a very small change in ANS fluorescence between 0 minute sample and 10 minute sample and it was 46.7 ± 17.6 a.u. The result is not surprising. Pepsin is a digestive enzyme in the stomach and the pH of the human stomach lumen is pH 1.5 to 3.5. A working pH range of pepsin is pH 1–4 and the optimum pH is around pH 2.5. At the condition, pH 2 with 150 mM NaCl and 65 °C, that we used, the structural change of pepsin should be affected by the temperature only, not by pH.

In this research, two fluorescent dyes, ThT and ANS, have been applied to characterize protein denaturation and protein aggregation. ANS is weakly fluorescent in hydrophilic environment such as water; however, its intensity is increased in hydrophobic environment such as nonpolar solvents^50,51^. ANS enhances fluorescence signals as it binds to hydrophobic regions on the protein surface^52,53^. Therefore, the ANS method has been used to detect transient states in protein denaturation. The fluorescent properties of ThT have also been well studied. ThT is used to investigate the formation of amyloid fibrils, as ThT does not bind to proteins in the folded, completely unfolded and partially folded or denaturated states of the type of a melted globule, as well as with amorphous aggregates of proteins^54,55^. Both amyloid fibrils and amorphous aggregates enhance the signal of light scattering; however, only amyloid fibrils increase the ThT fluorescence^54,55^. In protein aggregation, unfolded proteins could be formed by unfavorable environments, and the unfolded proteins are prone to form amorphous aggregates or fibrils via inappropriate interaction with other proteins since hydrophobic regions are exposed to the hydrophilic cell environment. For the protein aggregation in this research, both ANS and ThT fluorescence were monitored to understand the kinetics of protein denaturation and amyloid fibril formation.

**Figure 2:**
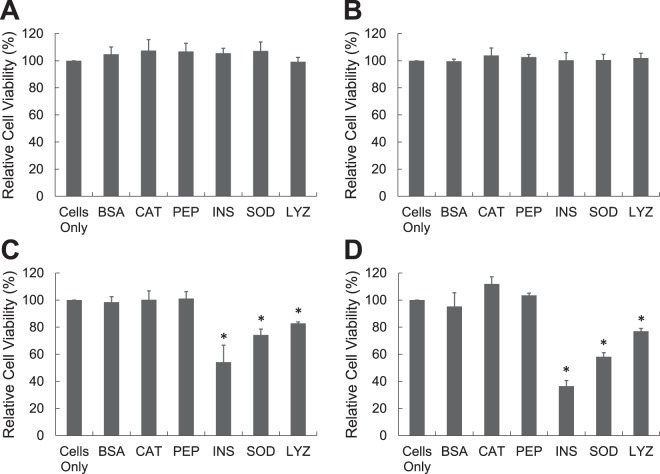
Cellular toxicity of protein aggregates was measured by MTT test. Protein (1 mg/mL) was prepared at pH 2 (150 mM NaCl) and 65 °C and it was neutralized prior to the addition to the PC 12 cells. Protein was incubated for (**A**) 10 minutes, (**B**) 20 minutes, (**C**) 30 minutes, and (**D**) 120 minutes [BSA: Bovine Serum Albumin, CAT: Catalase, PEP: Pepsin, INS: Insulin, SOD: Superoxide dismutase, LYZ: Lysozyme].

In Fig. 2, aliquots of six proteins (1 mg/mL) at pH 2 with 150 mM NaCl and 65 °C were withdrawn at 10, 20, 30, and 120 minutes, neutralized to pH 7.4, and added to PC-12 cells (ATCC^®^ CRL-1721^™^) that was a cell line derived from a pheochromocytoma of the rat adrenal medulla. After 24 h of incubation, cellular viability of protein aggregates was measured by the reduction of MTT (3-(4,5-Dimethylthiazol-2-yl)-2,5-Diphenyltetrazolium Bromide). Fresh or aged BSA, catalase, and pepsin did not induce any cytotoxicity to PC-12 cells. There was no statistical difference in MTT reduction between cells only in medium and cells treated with fresh or aged BSA, catalase, and pepsin. Other three proteins, lysozyme, insulin, and SOD, were not toxic when they were fresh; however they induced the cytotoxicity when incubated for more than 30 minutes. For insulin, protein for 30 minute incubation caused 45.8 ± 12.6% toxicity to PC-12 cells and insulin for 120 minute incubation increased the cytotoxicity to 63.6 ± 4.2%.

**Figure 3:**
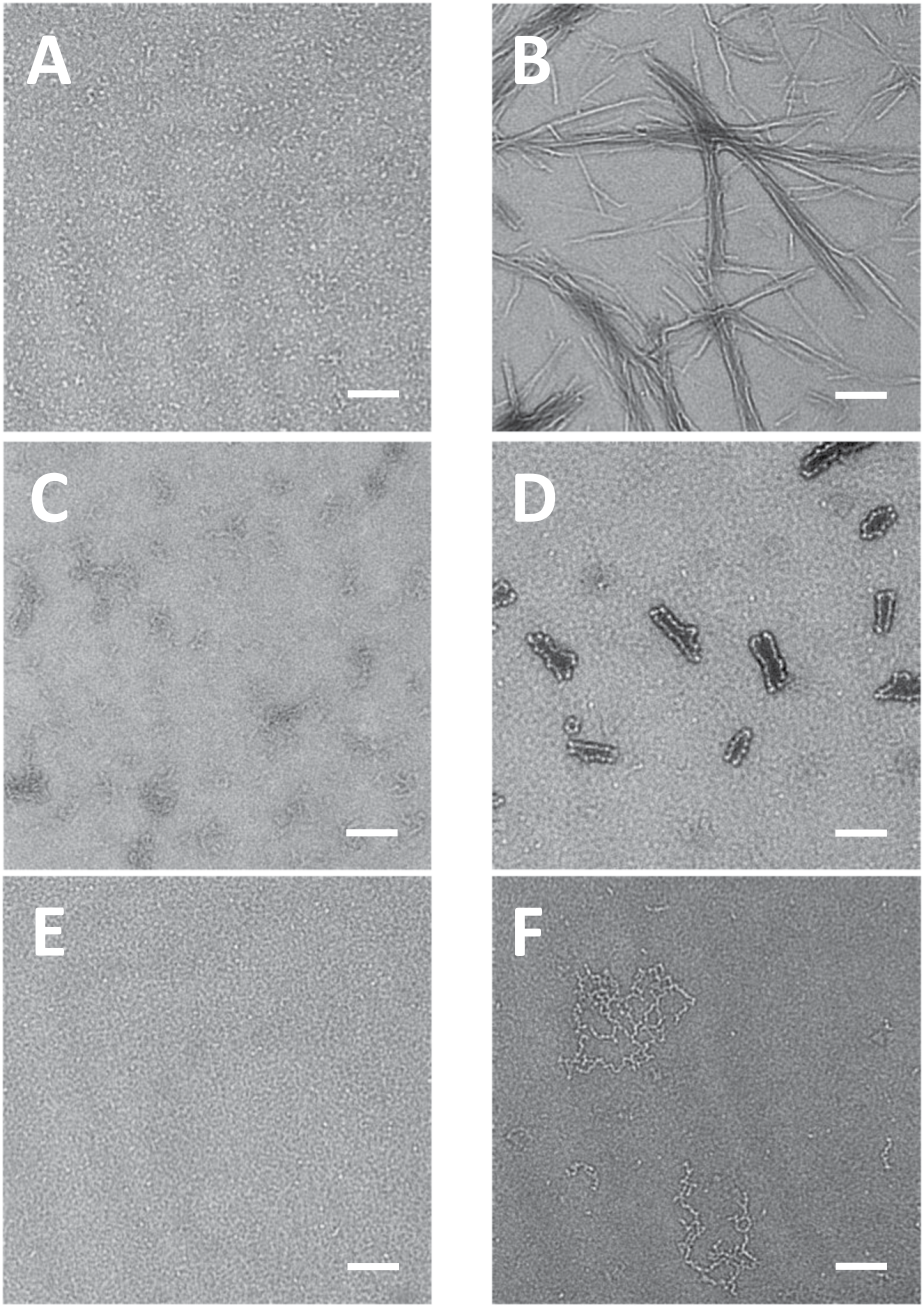
Morphology of protein aggregates was imaged by transmission electron microscopy (TEM). Proteins (1 mg/mL) were incubated at pH 2 (150 mM NaCl) and 65 °C for 120 minutes. (**A**) BSA, (**B**) Insulin, (**C**) Catalase, (**D**) Superoxide dismutase (SOD), (**E**) Pepsin, and (**F**) Lysozyme. Samples were negatively stained using 2% aqueous ammonium molybdate. Scale bar represents 100 nm.

Transmission electron micrograph (TEM) was used to visualize protein species formed at pH 2 and 65 °C for 2 hours. In Fig. 3, the morphological structures of BSA (Fig. 3A), catalase (Fig. 3C), and pepsin (Fig. 3E) at pH 2 and 65 °C for 2 hours were not different from those of fresh proteins. Although BSA (Fig. 3A) and catalase (Fig. 3C) did not produce fibrils, the denaturation of proteins resulted in seed- or protofibril- types of compounds. Due to the optimum pepsin activity at pH 1.5–1.6, the denaturation kinetics of pepsin might have been very slow in this condition (Fig. 3E), or pepsin was stable thermodynamically. Insulin (Fig. 3B) at pH 2 and 65 °C for 2 hours formed protofibrils or fibrils. The width of single protofibrils (n = 33) was 7.7 ± 1.8 nm. The length of most protofibrils varied from 161.0 nm to 638.9 nm. Protofibrils were arranged side by side to produce fibrils. In the same condition, SOD (Fig. 3D) and lysozyme (Fig. 3F) also formed protein aggregates. In Fig. 3D, misfolding of SOD initiated the formation of the seed for aggregation. The seed (n = 33) had uniform spherical shape with diameter of 9.6 ± 1.8 nm. The seeds elongated together and they became hairpin-like protofibrils. The oligomeric lysozyme protofibrils were also found in Fig. 3F. The width of single protofibrils (n = 33) was 8.5 ± 1.1 nm.

Based on Figs. 1–3, it is clear that insulin (1 mg/mL) at pH 2 and 65 °C for 120 minutes forms fibrils that are toxic to PC-12 cells. Fresh insulin that is mainly composed of insulin monomers is not toxic to PC-12 cells. The cellular toxicities of insulin at the time points of 10 minutes and 30 minutes are less than that of insulin fibrils at 120 minutes. In this result, there is no clear evidence that intermediates of insulin are more toxic than fibrils. However, the cellular toxicity of oligomeric β-amyloid (Aβ), another pathogenic protein for Alzheimer’s disease, has been studied relatively well. Soluble oligomeric forms of Aβ, termed amyloid-derived diffusible ligands (ADDLs) are known to be toxic species^56,57^. Cytotoxicity of Aβ may be maximal for the oligomers of intermediate size^58^. From monomer or dimer, cytotoxicity increased with oligomer size; however, cytotoxicity decreased with size when Aβ oligomers are bigger than 12-mers or higher^59,60^. In this research, other two pathogenic proteins, SOD and lysozyme, at pH 2 and 65 °C for 120 minutes forms protofibrils or premature fibrils (Fig. 3D,F) that induce cellular toxicity; however, they are less toxic than insulin fibrils. BSA and pepsin do not form any oligomers or fibrils at pH 2 and 65 °C for 120 minutes (Figs. 3A,E). In the same condition, there are small species in catalase (Fig. 3C); however, those small species are also shown in fresh catalase, which may result from the denaturation of catalase. BSA, catalase, and pepsin in this condition do not cause any cellular toxicity. Figures 1–3 demonstrate that cytotoxicity of proteins depends on the size of aggregates or fibrils as well as the type of proteins.

**Figure 4:**
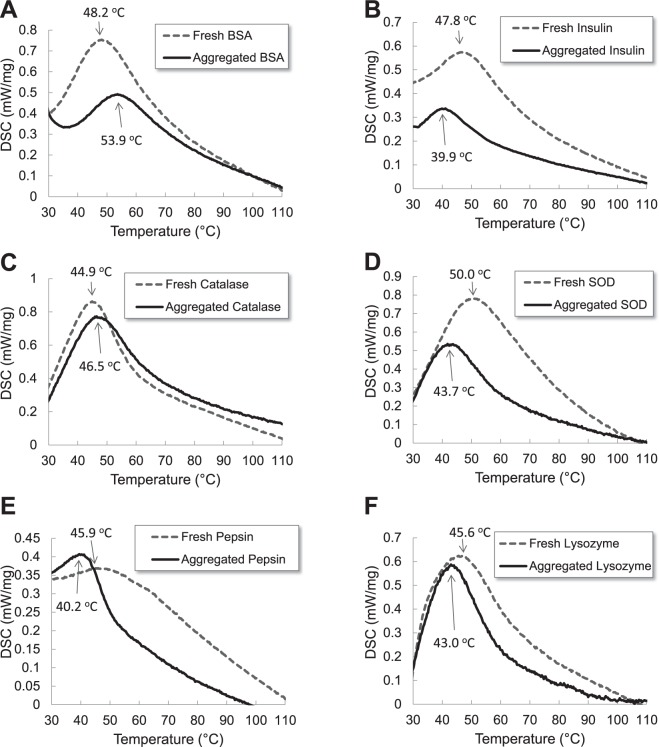
Differential scanning calorimetry (DSC) thermogram of fresh proteins and aged proteins (1 mg/mL) at pH 2 (150 mM NaCl) and 65 °C for 120 minutes. Fresh proteins (gray dotted line) and aged proteins (black solid line) at pH 2 and 65 °C for 120 minutes (**A**) BSA, (**B**) Insulin, (**C**) Catalase, (**D**) Superoxide dismutase (SOD), (**E**) Pepsin, and (**F**) Lysozyme.

Proteins in the physiological condition are thermodynamically stable and they are in a low energy state. Based on the state of protein folding, thermodynamic stability of proteins will change. Differential scanning calorimetry (DSC) is one of useful tools to measure thermal denaturation of proteins. In Fig. 4, thermal stabilities of six proteins were studied by DSC. Fresh proteins as folded proteins were used for control groups in the DSC analysis. Aged proteins (1 mg/mL) were also prepared at pH 2 (150 mM NaCl) and 65 °C for 120 minutes and the protein solution was freeze-dried. DSC thermogram of all powder proteins showed one thermal transition between 40 °C and 55 °C. Aged samples of lysozyme, insulin, SOD, and pepsin had low thermal transition compared to fresh samples. Other two proteins, BSA and catalase, demonstrated opposite thermal transition. Aged samples of BSA and catalase had high thermal transition compared to fresh samples. In the protein aggregation experiment, all samples were incubated at 65 °C that was above thermal transition temperatures of all proteins^61,62^. During the 2 hour incubation time, all proteins were denatured; however, protein denaturation of BSA and catalase was reversible as shown in TEM images. Reversible proteins were thermodynamically stable and thermal transitions of aged BSA and catalase were higher than those of fresh proteins. However, denaturation of other proteins, lysozyme, insulin, and SOD in this condition was irreversible and unfolded proteins were not refolded. Aged samples of lysozyme, insulin, and SOD were thermodynamically unstable, and thermal transitions of aged samples were lower than those of fresh proteins. All pathogenic proteins, lysozyme, insulin, and SOD, became thermodynamically unstable when they were incubated at 65 °C for 2 hours. Pre-fibrils, oligomers, fibrils or aggregates of pathogenic proteins had low thermal transitions or stabilities compared to fresh samples. Due to the low stability of pre-fibrils and oligomers, they assemble further to be more ordered structure or bigger species^63^. The unstable species can also interact with cell membrane, destabilize them, and impair membrane-bound proteins^64–66^. A series of processes induce the cellular toxicity^63^.

**Figure 5:**
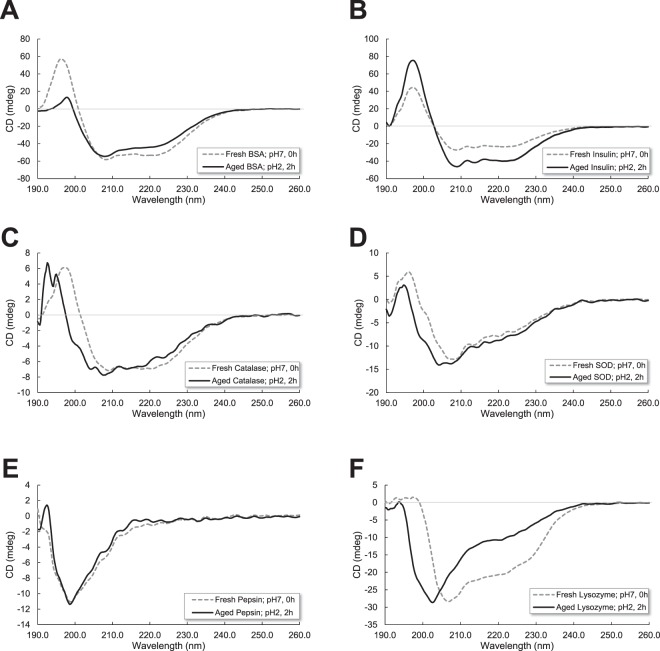
Secondary structure of protein aggregates was obtained by circular dichroism (CD). Proteins (1 mg/mL) were incubated at pH 2 (150 mM NaCl) and 65 °C for 120 minutes or 2 hours. Fresh proteins (gray dotted line) and proteins at pH 2 and 65 °C for 120 minutes or 2 hours (black solid line) (**A**) BSA, (**B**) Insulin, (**C**) Catalase, (**D**) Superoxide dismutase (SOD), (**E**) Pepsin, and (**F**) Lysozyme.

Circular dichroism (CD) spectroscopy has been used to examine secondary structure changes of six proteins during aggregation. As seen in Fig. 5, fresh proteins were a mixture of α-helix, β-sheet, turn, and random coil. Although changes in the CD spectra were observed when proteins were incubated at pH 2 and 65 °C for 2 hours, all six proteins did not indicate a significant change in the basic secondary structure elements from the deconvolution of CD spectra in Table 1.

**Table 1:** Percentage of secondary structure fractions for proteins at fresh (pH 7.0 for 0 hours) and aged (pH 2.0 for 2 hours) conditions.

Protein	Treatment	Secondary Structure			
		α-helix (%)	β-sheet (%)	Turn (%)	Others (%)
BSA	Fresh pH 7, 0 h	38.0	20.7	1.6	39.7
	Aged pH 2, 2 h	38.5	22.5	2.5	36.4
Catalase	Fresh pH 7, 0 h	3.3	37.9	14.1	44.8
	Aged pH 2, 2 h	3.4	35.7	15.5	45.4
Pepsin	Fresh pH 7, 0 h	0	42.8	14.4	42.8
	Aged pH 2, 2 h	0	42.1	14.9	43.0
Insulin	Fresh pH 7, 0 h	35.3	10.7	8.0	46.1
	Aged pH 2, 2 h	50.2	7.4	1.7	40.8
SOD	Fresh pH 7, 0 h	10.5	27.4	16.2	46.0
	Aged pH 2, 2 h	11.5	22.8	17.4	48.3
Lysozyme	Fresh pH 7, 0 h	24	18.2	15.6	42.2
	Aged pH 2, 2 h	12.1	18.8	16.9	52.1

The data were generated by deconvolution of CD spectra in Fig. 5.

Protein secondary structural information can be derived from CD signals. Two negative bands at 222 nm and 208 nm and a positive band at 193 nm are characteristic α-helix CD spectra^67^. The two negative bands arise from π–π* and n–π* transitions in the amide groups. Negative bands at 218 nm and positive bands at 195 nm are well-defined antiparallel β-sheets^68^. The conversion of the secondary structure into a predominantly antiparallel β-sheet is a pathologic process of pathogenic proteins^69^. In Table 1, only BSA and lysozyme increased β-sheet structure in the pH 2 and 2 hour condition. Other pathogenic proteins, insulin and SOD, increased α-helix structure in the pH 2 and 2 hour condition. Overall, pathogenic proteins formed more ordered structures such as α-helix and β-sheet in the pH 2 and 2 hour condition; however, the differences are not drastic.

In order to understand the effect of protein concentration on the protein aggregation, we increased the protein concentration of six proteins to 20 mg/mL and we repeated experiments of protein aggregation kinetics, cytotoxicity, and TEM imaging. Protein concentration is one of key factors in protein aggregation^14^. Proteins increase the chance of collision at higher concentrations, which enhances the formation of protein aggregates^16^.

**Figure 6:**
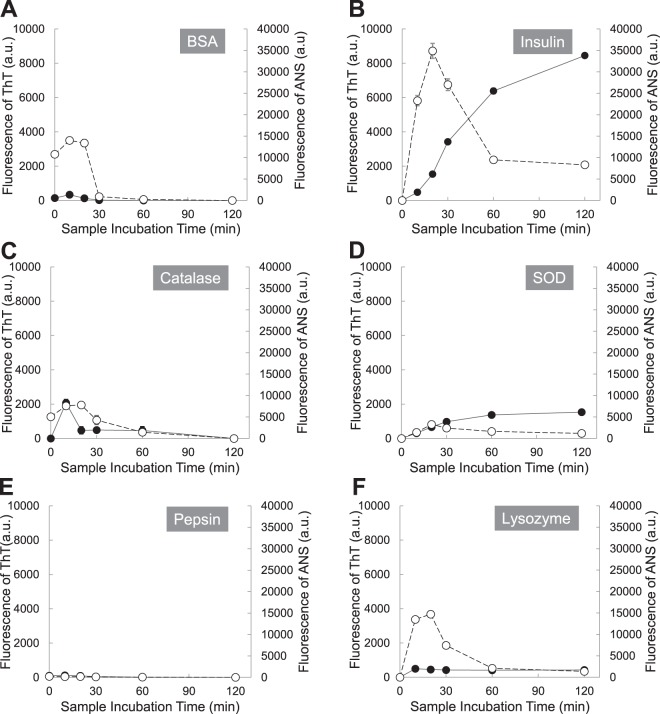
Protein (20 mg/mL) aggregation kinetics at pH 2 (150 mM NaCl) and 65 °C was monitored by fluorescence of Thioflavin T (ThT) and 8-Anilinonaphthalene-1-sulfonic acid (ANS). ThT fluorescence (black circles) and ANS fluorescence (white circles); (**A**) BSA, (**B**) Insulin, (**C**) Catalase, (**D**) Superoxide dismutase (SOD), (**E**) Pepsin, and (**F**) Lysozyme.

Here, six proteins (20 mg/mL) were incubated at pH 2 with 150 mM NaCl and 65 °C, and the aliquots were withdrawn at the following time points (0, 10, 20, 30, 60 and 120 minutes). In Fig. 6, all proteins increased ThT fluorescence emission without a lag time; however, BSA, catalase, and pepsin reached the top of ThT fluorescence in 10 minutes and the fluorescence were diminished with time after 10 minutes. Other proteins, lysozyme, insulin, and SOD, steadily increased ThT fluorescence emission and they reached the saturation level in 30 minutes. The ANS fluorescence of all proteins was increased up to 10 minutes, confirming the presence of molten globular intermediates or denaturation of proteins.

**Figure 7:**
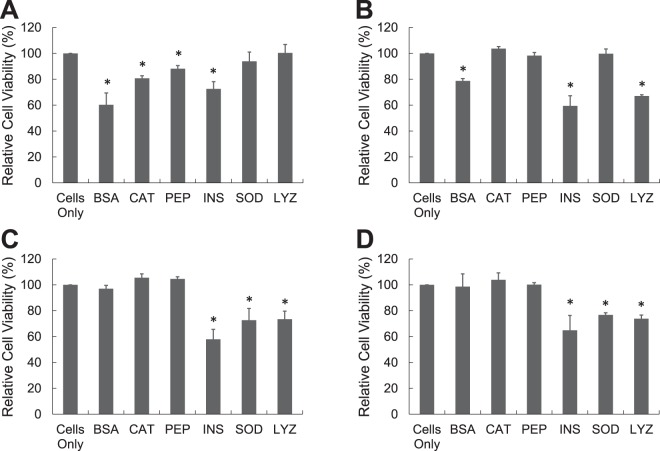
Cellular toxicity of protein aggregates was measured by MTT test. Protein (20 mg/mL) was prepared at pH 2 (150 mM NaCl) and 65 °C and it was neutralized prior to the addition to the PC 12 cells. Protein was incubated for (**A**) 10 minutes, (**B**) 20 minutes, (**C**) 30 minutes, and (**D**) 60 minutes [BSA: Bovine Serum Albumin, CAT: Catalase, PEP: Pepsin, INS: Insulin, SOD: Superoxide dismutase, LYZ: Lysozyme].

In Fig. 7, aliquots of six proteins (20 mg/mL) at pH 2 with 150 mM NaCl and 65 °C were withdrawn at 0, 10, 30, and 120 minutes, neutralized to pH 7.4, and added to PC-12. BSA, catalase, and pepsin samples at 10 minutes caused 39.7 ± 9.1%, 19.3 ± 2.0%, and 11.8 ± 2.4% toxicities to PC-12 cells respectively. Interestingly toxicities of aged BSA, catalase, and pepsin disappeared when they were incubated longer than 10 minutes. Other three proteins, lysozyme, insulin, and SOD, induced the cytotoxicity when incubated for more than 30 minutes, which was similar to lower concentration (1 mg/mL) of proteins.

**Figure 8:**
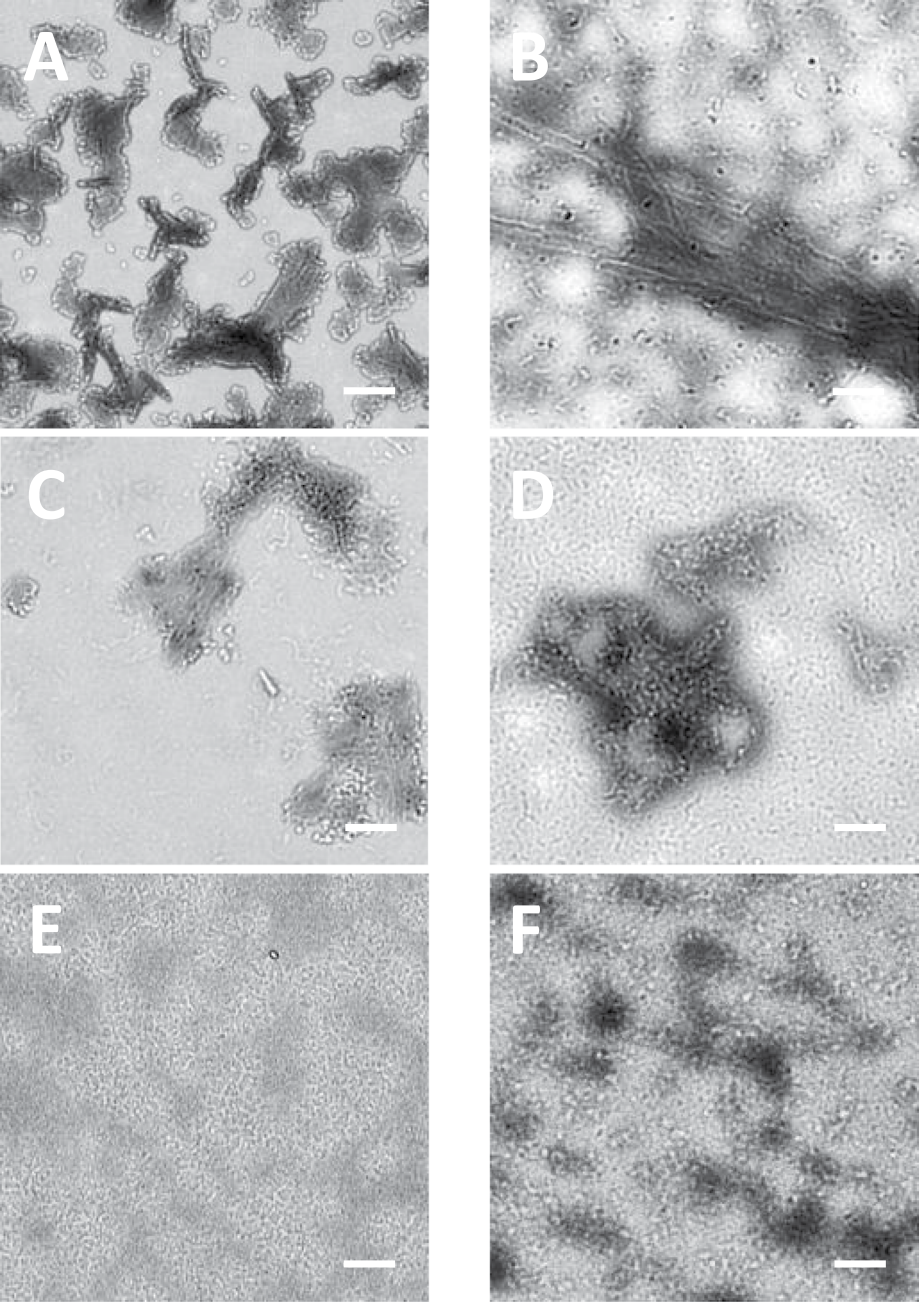
Morphology of protein aggregates was imaged by TEM. Proteins (20 mg/mL) were incubated at pH 2 (150 mM NaCl) and 65 °C for 120 minutes. (**A**) BSA, (**B**) Insulin, (**C**) Catalase, (**D**) Superoxide dismutase (SOD), (**E**) Pepsin, and (**F**) Lysozyme. Samples were negatively stained using 2% aqueous ammonium molybdate. Scale bar represents 100 nm.

Transmission electron micrograph (TEM) was used again to visualize the higher concentration of protein species formed at pH 2 and 65 °C for 2 hours. In Fig. 8, all proteins except pepsin showed larger protein aggregates. All proteins formed spherical species in this condition and the sizes of spherical species were between 8 nm and 12 nm in diameter. Spherical species were basic unit of protofibrils and they elongated into protofibrils or fibrils via doughnut shapes. The diameter of protofibrils or fibrils was around 10 nm that was the characteristic diameter of fibrils.

In Figs. 6 and 7, even non-pathogenic proteins such as BSA and catalase, induced cellular toxicities at a higher concentration (20 mg/mL). In the incubation condition of pH 2 and 65 °C, all proteins are denatured and the fluorescence of ANS was enhanced as a result. In both 1 mg/mL and 20 mg/mL concentrations, non-pathogenic proteins increased the fluorescence of ANS less than 10 minutes. However, the ThT fluorescence of BSA and catalase at 20 mg/mL was about 10-fold higher than that of BSA and catalase at 1 mg/mL. It is clear that BSA and catalase at 20 mg/mL have more ordered structure than at 1 mg/mL since thioflavin T increases the fluorescence signal when it binds to oligomeric or fibrillar proteins with a high β-sheet or α-helix content. However, the toxic species are not clearly understood. One possible explanation is that BSA or catalase may produce same toxic species at both 1 mg/mL and 20 mg/mL, and only 20 mg/mL concentration generates enough amounts of toxic species to cause cellular toxicity. Another explanation is that BSA or catalase at 20 mg/mL makes different species from BSA or catalase at 1 mg/mL, and those species could be an ordered form such as micelles that induce cellular toxicity. Interestingly BSA or catalase at 20 mg/mL caused the cellular toxicities at the only early incubation time. As time went, BSA or catalase had less ordered structures and they became less hydrophobic since both ThT and ANS signals were reduced. Unstructured hydrophilic compounds may be relatively less toxic than ordered hydrophobic structures.

In summary, we investigated the cellular toxicity of pathogenic or non-pathogenic protein aggregates. Three proteins, lysozyme, insulin, superoxide dismutase, were selected as pathogenic proteins, and other three proteins, catalase, SOD, pepsin, were chosen as non-pathogenic proteins. Six proteins (1 mg/mL) were incubated at pH 2 with 150 mM NaCl and 65 °C, and three pathogenic proteins, lysozyme, insulin, and SOD, enhanced ThT fluorescence emission and they reached the saturation level in 30–60 minutes. Based on TEM images and MTT test, fibrils or aggregates of three pathogenic proteins induced the cellular toxicity. Other three non-pathogenic proteins, BSA, catalase, and pepsin were stable, and they didn’t cause the cellular toxicity in this condition. According to DSC analysis, fibrils or aggregates of pathogenic proteins had low thermal transition compared to fresh samples. Low thermal stability is associated with the cellular toxicity. At a higher concentration (20 mg/mL), even non-pathogenic proteins cause cellular toxicity in the early incubation time. Pathogenic proteins induced the cellular toxicity like a lower concentration (1 mg/mL). All results in this research would be beneficial to understand similarities and differences in protein aggregation kinetics, cellular toxicity, and morphological structures of pathogenic and non-pathogenic proteins.
